# Association Between the Dialysate Bicarbonate and the Pre-dialysis Serum Bicarbonate Concentration in Maintenance Hemodialysis: A Retrospective Cohort Study

**DOI:** 10.1177/20543581241256774

**Published:** 2024-05-30

**Authors:** Amber O. Molnar, Lauren Killin, Sarah Bota, Eric McArthur, Stephanie N. Dixon, Amit X. Garg, Claire Harris, Stephanie Thompson, Karthik Tennankore, Peter G. Blake, Clara Bohm, Jennifer MacRae, Samuel A. Silver

**Affiliations:** 1 Division of Nephrology, Department of Medicine, McMaster University, Hamilton, ON, Canada; 2 Institute for Clinical Evaluative Sciences, London, ON, Canada; 3 Department of Health Research Methods, Evidence, and Impact, McMaster University, Hamilton, ON, Canada; 4 Population Health Research Institute, McMaster University/Hamilton Health Sciences, ON, Canada; 5 St. Joseph’s Healthcare Hamilton, Hamilton, ON, Canada; 6 Lawson Health Research Institute, London Health Sciences Centre, ON, Canada; 7 Division of Nephrology, Department of Medicine, Western University, London, ON, Canada; 8 Department of Epidemiology, Western University, London, ON, Canada; 9 Division of Nephrology, Department of Medicine, The University of British Columbia, Vancouver, Canada; 10 Division of Nephrology, Department of Medicine, University of Alberta, Edmonton, Canada; 11 Division of Nephrology, Department of Medicine, Nova Scotia Health Authority, Halifax, Canada; 12 Division of Nephrology, Department of Medicine, University of Manitoba, Winnipeg, Canada; 13 Division of Nephrology, Department of Medicine, University of Calgary, AB, Canada; 14 Division of Nephrology, Department of Medicine, Queen’s University, Kingston, ON, Canada

**Keywords:** dialysis, hemodialysis, metabolic acidosis

## Abstract

**Background:**

It is unclear whether the use of higher dialysate bicarbonate concentrations is associated with clinically relevant changes in the pre-dialysis serum bicarbonate concentration.

**Objective:**

The objective is to examine the association between the dialysate bicarbonate prescription and the pre-dialysis serum bicarbonate concentration.

**Design:**

This is a retrospective cohort study.

**Setting:**

The study was performed using linked administrative health care databases in Ontario, Canada.

**Patients:**

Prevalent adults receiving maintenance in-center hemodialysis as of April 1, 2020 (n = 5414) were included.

**Measurements:**

Patients were grouped into the following dialysate bicarbonate categories at the dialysis center-level: individualized (adjustment based on pre-dialysis serum bicarbonate concentration) or standardized (>90% of patients received the same dialysate bicarbonate concentration). The standardized category was stratified by concentration: 35, 36 to 37, and ≥38 mmol/L. The primary outcome was the mean outpatient pre-dialysis serum bicarbonate concentration at the patient level.

**Methods:**

We examined the association between dialysate bicarbonate category and pre-dialysis serum bicarbonate using an adjusted linear mixed model.

**Results:**

All dialysate bicarbonate categories had a mean pre-dialysis serum bicarbonate concentration within the normal range. In the individualized category, 91% achieved a pre-dialysis serum bicarbonate ≥22 mmol/L, compared to 87% in the standardized category. Patients in the standardized category tended to have a serum bicarbonate that was 0.25 (95% confidence interval [CI] = −0.93, 0.43) mmol/L lower than patients in the individualized category. Relative to patients in the 35 mmol/L category, patients in the 36 to 37 and ≥38 mmol/L categories tended to have a serum bicarbonate that was 0.70 (95% CI = −0.30, 1.70) mmol/L and 0.87 (95% CI = 0.14, 1.60) mmol/L higher, respectively. There was no effect modification by age, sex, or history of chronic lung disease.

**Limitations:**

We could not directly confirm that all laboratory measurements were pre-dialysis. Data on prescribed dialysate bicarbonate concentrations for individual dialysis sessions were not available, which may have led to some misclassification, and adherence to a practice of individualization could not be measured. Residual confounding is possible.

**Conclusions:**

We found no significant difference in the pre-dialysis serum bicarbonate concentration irrespective of whether an individualized or standardized dialysate bicarbonate was used. Dialysate bicarbonate concentrations ≥38 mmol/L (vs 35 mmol/L) may increase the pre-dialysis serum bicarbonate concentration by 0.9 mmol/L.

## Abrégé

**Contexte:**

On ignore si des concentrations plus élevées de bicarbonate dans le dialysat sont associées à des changements cliniquement significatifs dans le taux de bicarbonate sérique prédialyse.

**Objectif:**

Examiner l’association entre la prescription de bicarbonate du dialysat et le taux de bicarbonate sérique prédialyse.

**Conception:**

Étude de cohorte rétrospective.

**Cadre:**

Étude réalisée en Ontario (Canada) à partir des données administratives de santé.

**Sujets:**

Ont été inclus les adultes prévalents qui recevaient une hémodialyse chronique en centre le 1^er^ avril 2020 (n=5 414).

**Mesures:**

Les sujets ont été regroupés dans les catégories suivantes de concentration en bicarbonate dans le dialysat utilisée dans leur unité de dialyse: individualisée (ajustée selon le taux de bicarbonate sérique prédialyse) ou normalisée (même concentration pour >90% des sujets). La catégorie « standardisée » a été stratifiée selon la concentration: 35 mmol/L, 36 à 37 mmol/L et ≥38 mmol/L. Le principal critère d’évaluation était le taux moyen de bicarbonate sérique prédialyse en ambulatoire au niveau du patient.

**Méthodologie:**

Nous avons examiné l’association entre la catégorie de concentration en bicarbonate du dialysat et le taux de bicarbonate sérique prédialyse à l’aide d’un modèle linéaire mixte corrigé.

**Résultats:**

Pour toutes les catégories de concentration en bicarbonate du dialysat, le taux moyen de bicarbonate sérique prédialyse était dans la plage normale. Dans la catégorie « individualisée », 91% des sujets avaient un taux de bicarbonate sérique prédialyse de ≥22 mmol/L, comparativement à 87% dans la catégorie « standardisée ». Les patients de la catégorie « standardisée » tendaient à avoir un taux de bicarbonate sérique de 0,25 mmol/L (IC 95%: -0,93 à 0,43) inférieur à celui des patients de la catégorie « individualisée ». Comparé aux patients de la catégorie 35 mmol/L, les patients des catégories 36 à 37 mmol/L et ≥38 mmol/L tendaient respectivement à avoir un taux de bicarbonate sérique de 0,70 mmol/L (IC 95%: -0,30 à 1,70) et de 0,87 mmol/L (IC 95%: 0,14 à 1,60) plus élevé. L’âge, le sexe ou les antécédents de maladie pulmonaire chronique n’ont pas semblé modifier l’effet.

**Limites:**

Il n’a pas été possible de confirmer directement que toutes les mesures de laboratoire avaient été effectuées avant la dialyse. Les données sur les concentrations de bicarbonate prescrites pour les séances de dialyse individuelles n’étaient pas disponibles, ce qui peut avoir conduit à une classification erronée. De plus, l’observance d’une pratique d’individualisation n’a pas pu être mesurée. Une confusion résiduelle est possible.

**Conclusion:**

Nous n’avons observé aucune différence significative dans les taux de bicarbonate sériques prédialyse, qu’on ait utilisé une concentration individualisée ou standardisée de bicarbonate dans le dialysat. L’utilisation d’un dialysat à ≥38 mmol/L (c. 35 mmol/L) de bicarbonate peut entraîner une hausse de 0,9 mmol/L du taux de bicarbonate sérique prédialyse.

## Introduction

A key function of hemodialysis is to correct the metabolic acidosis of kidney failure, which is accomplished by adding bicarbonate to the dialysate. Guidelines recommend maintaining a pre-dialysis serum bicarbonate concentration ≥22 mmol/L, but this is based on weak evidence.^1,2^ Large observational studies demonstrate an association between metabolic acidosis and increased mortality risk.^3,4^ Small trials suggest that reversal of metabolic acidosis (increase in serum bicarbonate ~5 mmol/L) has positive effects on nutrition, muscle mass, and bone metabolism.^5-9^ Hence, it is common in North America to prescribe dialysate bicarbonate concentrations ≥35 mmol/L.^2,10^ However, prior studies suggest that the dialysate bicarbonate concentration may have a negligible effect on the pre-dialysis serum bicarbonate concentration,^11-15^ and observational evidence shows that the use of high dialysate bicarbonate concentrations is associated with increased mortality.^10^

There exists a competing balance of reversing metabolic acidosis while avoiding the potential harms of metabolic alkalosis.^16^ Practice patterns with respect to dialysate bicarbonate are quite variable; some centers use a standardized approach (nearly all patients in a given dialysis center receive the same concentration of dialysate bicarbonate), whereas other centers use an individualized approach (a patient’s dialysate bicarbonate concentration is adjusted so that they achieve a certain pre-dialysis serum bicarbonate concentration).^10^ Furthermore, the dialysate bicarbonate concentration that is routinely prescribed to patients varies between 32 and 40 mmol/L.^10^

To better understand the association between the concentrations of dialysate bicarbonate and pre-dialysis serum bicarbonate in patients receiving maintenance hemodialysis, we performed a population-based retrospective cohort study using routinely collected health care data from 25 hemodialysis programs in Ontario, Canada. The practice variation across regional dialysis programs in Ontario allowed us to examine these associations. We hypothesized that there would be no significant association between the dialysate bicarbonate prescription and pre-dialysis serum bicarbonate concentration.

## Materials and Methods

### Design and Setting

We conducted a population-based retrospective cohort study with administrative health care databases linked using unique encoded identifiers and analyzed at ICES (ices.on.ca/) in Ontario, Canada. The study was conducted according to a pre-specified protocol. The use of data in this project was authorized under section 45 of Ontario’s Personal Health Information Protection Act, which did not require review by a Research Ethics Board. The reporting of this study follows the Reporting of studies Conducted using Observational Routinely collected health Data (RECORD) guidelines for observational studies (Supplemental Appendix A).^17^

### Data Sources

Patients receiving in-center maintenance hemodialysis and dialysis-related baseline characteristics were identified using the Ontario Renal Reporting System (ORRS) database. The Canadian Organ Replacement Register (CORR), a federal government mandated end-stage kidney disease (ESKD) registry,^18^ was used to determine outpatient dialysis start date. The Ontario Laboratories Information System (OLIS) contains laboratory results from hospital, community, and public health labs across Ontario and was used to determine the primary outcome of pre-dialysis serum bicarbonate and all secondary outcomes. Dialysate bicarbonate concentrations prescribed by regional dialysis programs across Ontario were determined by contacting dialysis unit directors. Full details on databases and codes used can be found in Supplemental Appendix B.

### Study Cohort

We included prevalent adults receiving maintenance in-center hemodialysis at the same regional dialysis program for at least 120 days as of April 1, 2020 (index date). Patients receiving in-center short daily dialysis or nocturnal hemodialysis at baseline were excluded as they often receive a lower dialysate bicarbonate concentration than patients on a conventional thrice weekly dialysis schedule. Patients from 2 Ontario regional dialysis programs with no laboratory data available through OLIS were excluded.

We assessed comorbidities in the 3 years prior to the index date, laboratory measures and health care utilization in the 1 year prior to the index date, and medications in the 120 days prior to the index date for patients over 65 years of age, receiving home care, or in social assistance programs eligible for coverage under the Ontario Drug Benefit plan.

### Dialysate Bicarbonate Categories and Outcomes

Dialysate bicarbonate practices were categorized at the dialysis program level. Programs were grouped into the dialysate bicarbonate categories of individualized (adjustment to the dialysate bicarbonate concentration based on the pre-dialysis serum bicarbonate) or standardized (>90% of patients at the dialysis regional program received the same dialysate bicarbonate concentration). The dialysis programs with a standardized approach were grouped into the categories of 35 mmol/L, 36 to 37 and ≥38 mmol/L (no standardized programs in Ontario used a concentration <35 mmol/L). The primary outcome was the mean outpatient pre-dialysis serum bicarbonate concentration over the follow-up period at the patient level. Serum bicarbonate values that were <10 or >45 mmol/L were excluded due to biological implausibility. Secondary outcomes were mean outpatient pre-dialysis serum potassium, total calcium, and albumin concentrations. Total calcium values were corrected for the most recent serum albumin value within the prior 30 days using the formula of corrected calcium concentration = measured total calcium concentration in mmol/L + (0.02 * [40 g/L − serum albumin concentration in g/L]).^19^

Patients were followed for 1 year (until March 31, 2021) and were censored upon death, 30 days following transfer to a new dialysis regional program, permanent discontinuation of in-center hemodialysis (recovery of kidney function or withdrawal from dialysis), 30 days after transfer to a home dialysis modality or in-center short daily or nocturnal dialysis, receipt of a kidney transplant, or emigration from the province (<0.1%).

### Statistical Analysis

We used standardized differences to compare baseline characteristics between dialysate bicarbonate categories (individualized vs standardized and standardized concentrations of 35 vs 36-37 and ≥38 mmol/L). Standardized differences ≥0.1 were considered a significant difference between categories.^20^ For each dialysate bicarbonate category, we determined, over the 1-year follow-up, the mean serum bicarbonate, potassium, corrected calcium and albumin, and the proportion of patients achieving a mean serum bicarbonate concentration ≥22 mmol/L, as recommended in some guidelines.^1,2^

We then examined the association of each dialysate bicarbonate category with the pre-dialysis serum bicarbonate concentration using a linear mixed model accounting for dialysis program clustering and controlling for longitudinal measurements (repeated observations at the individual patient level, accounting for the time since accrual of the measurements as a fixed effect). Serum bicarbonate was treated as a continuous outcome, and we assumed a linear relationship. The distribution of the dialysis center random effect was assumed to be normally distributed with a mean of 0 and variance of σ^2^. Time was included as a fixed effect in the model and modeled continuously. Patients with no outpatient serum bicarbonate measurements over the follow-up did not contribute to the model (n = 798). Individualized (referent) dialysate bicarbonate was compared to standardized. The standardized category was then divided based on dialysate bicarbonate concentration (35, 36-37, ≥38 mmol/L), and the individualized group (referent) was compared to each concentration category. The cohort was then restricted to patients in the standardized category, and 35 mmol/L (referent) was compared to 36-37 and ≥38 mmol/L. Models are presented as unadjusted and adjusted for age (continuous), sex, income quintile, rural status, vascular access, cause of ESKD, heart failure, peripheral vascular disease, chronic obstructive pulmonary disease (COPD), prior coronary artery bypass surgery (CABG), prior gastrointestinal bleed, Charlson Comorbidity Index, number of hospitalizations, number of emergency room visits, number of primary care physician visits, number of cardiologist visits, smoking status, body mass index (BMI), and baseline hemoglobin, potassium, corrected calcium, albumin, parathyroid hormone, and serum bicarbonate. For adjusted models, missing values for BMI and laboratory measures were imputed with multiple imputation using a fully conditional specification regression method. The linear mixed model analysis was repeated for the secondary outcomes of serum potassium, corrected calcium, and albumin. The primary analysis was conducted in the following pre-specified subgroups: age (<65, ≥65-80, >80), sex, and history of COPD. Patients with COPD were examined as a subgroup due to the potential impact of chronic lung disease with associated hypercapnia on serum bicarbonate. The primary analysis was repeated, with additional adjustment for medications (diuretics, calcium carbonate, sevelamer, proton pump inhibitors, statins, beta-blockers, angiotensin-converting enzyme inhibitors, angiotensin receptor blockers, oral anticoagulants, alpha blockers, calcium channel blockers, nitrates, hydralazine), in a cohort restricted to individuals eligible for Ontario Drug Benefit coverage (for which medication data were available). Two-sided *P*-values <.05 were considered significant. All analyses were completed using SAS version 9.4.

## Results

### Baseline Characteristics

Cohort selection is detailed in Supplemental Figure 1. From a total of 5652 prevalent patients receiving maintenance in-center hemodialysis as of April 1, 2020, we identified 5414 eligible patients. Patients in the individualized (n = 2159) and standardized (n = 3255) categories had similar baseline characteristics. Compared to the 35 mmol/L category (n = 2036), patients in the 36 to 37 mmol/L category (n = 324) were less likely to be female (36% vs 41%), more likely to have a history of heart failure (39% vs 33%), COPD (38% vs 28%) and CABG (6% vs 2%), and had a higher baseline serum bicarbonate (25.5 vs 24.0 mmol/L). Compared to the 35 mmol/L category, patients in the ≥38 mmol/L category (n = 895) had a higher baseline serum bicarbonate (26.4 vs 24.0 mmol/L) (Table 1).

**Table 1.** Baseline Characteristics.

	Individualized dialysate bicarbonate concentration (N = 2159)	Standardized dialysate bicarbonate concentration			
		Categories of 35, 36-37, and ≥38 mmol/L all together (N = 3255)	35 mmol/L (N = 2036)	36-37 mmol/L (N = 324)	≥38 mmol/L (N = 895)
Age	68 (14.3)	68 (14.1)	68 (13.9)	71 (13.7)^a^	66 (14.5)^a^
Female	884 (41)	1339 (41)	839 (41)	117 (36)^a^	383 (43)
Income quintile^b^					
1	701 (33)	1131 (35)	688 (34)	94 (29)^a^	349 (39)^a^
2	421 (20)	738 (23)	469 (23)	70 (22)	199 (22)
3	423 (20)	544 (17)	336 (17)	73 (23)^a^	135 (15)
4	347 (16)	489 (15)	315 (16)	53 (16)	121 (14)
5	267 (12)	353 (11)	228 (11)	34 (11)	91 (10)
Rural location^c^	149 (7)	444 (14)^d^	253 (11)	19 (6)^a^	172 (19)^a^
Time on dialysis, years	5.6 (6.5)	5.0 (5.5)	5.1 (5.7)	4.6 (4.6)	5.1 (5.5)
Dialysis access					
AVF	628 (29)	1162 (36)^d^	769 (38)	109 (34)	284 (32)^a^
AVG	115 (5)	99 (3)^d^	68 (3)	6 (2)	25 (3)
CVC	1403 (65)	1969 (61)	1199 (59)	209 (65)^a^	586 (66)^a^
Unknown	13 (0.6)	25 (0.8)	Imputed as CVC	Imputed as CVC	Imputed as CVC
Smoker at dialysis initiation	261 (12)	423 (13)	234 (12)	60 (19)^a^	129 (14)
BMI at dialysis initiation	29.2 (7.4)	29.1 (7.6)	28.7 (7.5)	30.1 (7.4)^a^	29.5 (7.9)^a^
Missing	421 (20)	453 (14)	316 (16)	26 (8)	111 (12)
Primary cause of kidney disease					
GN	287 (13)	389 (12)	255 (13)	32 (10)	102 (11)
PKD	82 (4)	140 (4)	82 (4)	17 (5)	41 (5)
Diabetes	756 (35)	1254 (39)	745 (37)	126 (39)	383 (43)
RVD	222 (10)	483 (15)^d^	324 (16)	47 (15)	112 (13)^a^
Drug-induced	25 (1)	62 (2)	28 (1)	10 (3)^a^	24 (3)
Other	787 (37)	927 (29)	602 (30)	92 (28)	232 (26)
Atrial fibrillation	400 (19)	670 (21)	427 (21)	74 (23)	169 (19)
Heart failure	729 (34)	1076 (33)	664 (33)	125 (39)^a^	287 (32)
Ischemic stroke	79 (4)	99 (3)	69 (3)	7 (2)	23 (3)
PVD	211 (10)	383 (12)	290 (14)	27 (8)^a^	66 (7)^a^
COPD	589 (27)	958 (29)	578 (28)	124 (38)^a^	256 (29)
Diabetes	1414 (66)	2186 (67)	1365 (67)	216 (67)	605 (68)
CABG	58 (3)	89 (3)	42 (2)	19 (6)^a^	28 (3)
GI bleed	230 (11)	344 (11)	189 (9)	38 (12)	117 (13)^a^
Charlson Comorbidity Index	5.3 (2.1)	5.3 (2.1)	5.3 (2.1)	5.6 (2.2)^a^	5.3 (2.1)
Major cancer	290 (13)	432 (13)	273 (13)	52 (16)	107 (12)
Primary care physician visits^e^	12.3 (19.7)	10.6 (15.7)^d^	10.1 (14.9)	11.4 (12.6)^a^	11.4 (18.2)
Cardiologist visits^e^	3.0 (5.6)	2.5 (4.2)^d^	2.5 (4.2)	2.2 (4.4)	2.6 (4.2)
Emergency department visits^e^	1.9 (2.7)	2.0 (3.0)	1.8 (2.6)	1.9 (2.4)	2.5 (3.8)^a^
Hospitalizations^e^	0.9 (1.3)	0.8 (1.3)	0.8 (1.2)	0.9 (1.4)^a^	1.0 (1.5)^a^
Diuretics^f^	643 (30)	1033 (32)	664 (33)	139 (43)^a^	230 (26)^a^
Oral sodium bicarbonate^f^	13 (0.6)	20 (0.6)	<6 (<0.3)^g^	0 (0)	15-19 (2)^g^
Proton pump inhibitors	903 (42)	1354 (42)	815 (40)	168 (52)^a^	371 (42)
Statins	1157 (54)	1722 (53)	1058 (52)	188 (58)^a^	476 (53)
Calcium carbonate	578 (27)	788 (24)	460 (23)	71 (22)	257 (29)^a^
Sevelamer	155 (7)	219 (7)	161 (8)	15 (5)^a^	43 (5)^a^
Oral anticoagulants	251 (12)	412 (13)	253 (12)	71 (22)^a^	88 (10)
Angiotensin-converting enzyme inhibitors	318 (15)	450 (14)	261 (13)	38 (12)	151 (17)^a^
Angiotensin receptor blockers	424 (20)	756 (23)	513 (25)	74 (23)	169 (19)^a^
Beta-blockers	1004 (47)	1529 (47)	939 (46)	175 (54)^a^	415 (46)
Alpha blockers	291 (14)	469 (14)	317 (16)	26 (8)^a^	126 (14)
Calcium channel blockers	929 (43)	1480 (46)	898 (44)	161 (50)^a^	421 (47)
Nitrates	167 (8)	280 (9)	163 (8)	37 (11)^a^	80 (9)
Hydralazine	170 (8)	192 (6)	106 (5)	38 (12)^a^	48 (5)
Hemoglobin^h^ (g/L)	108.1 (12.3)	108.7 (12.5)	108.4 (12.4)	110.6 (11.4)^a^	108.8 (13.2)
≥1 measurement available	1976 (92)	3063 (94)	1989 (98)	323 (100)	751 (84)
Serum bicarbonate (mmol/L)^h^	25.0 (3.8)	24.8 (3.6)	24.0 (3.3)	25.5 (2.9)^a^	26.4 (4.0)^a^
≥1 measurement available	1890 (88)	3104 (95)	1975 (97)	321 (99)	808 (90)
Serum calcium (mmol/L)^h,i^	2.3 (0.2)	2.3 (0.2)	2.3 (0.2)	2.4 (0.2)^a^	2.3 (0.2)^a^
≥1 measurement available	1885 (87)	3005 (92)	1974 (97)	320 (99)^g^	711 (79)
Serum potassium (mmol/L)^h^	4.6 (0.7)	4.6 (0.7)	4.7 (0.7)	4.7 (0.6)	4.5 (0.8)
≥1 measurement available	1965 (91)	3130 (96)	1982 (97)	321 (99)	827 (92)
Serum albumin^h^ (g/L)	37.4 (4.9)	36.7 (4.9)^d^	36.3 (4.8)	33.5 (4.0)^a^	38.8 (4.5)^a^
≥1 measurement available	1907 (88)	3115 (96)	1975 (97)	320 (99)	820 (92)
PTH^h^ (pmol/L)	69.8 (74.3)	55.9 (53.0)^d^	56.0 (54.0)	57.2 (50.8)	55.1 (51.5)
≥1 measurement available	1836 (85)	2916 (90)	1842 (91)	307 (95)	767 (86)

Continuous variables are reported as mean (standard deviation) unless otherwise specified. Categorical variables are reported as n (%). Standardized differences ≥0.1 were considered statistically significant.

a Standardized difference ≥0.1 compared to dialysate bicarbonate concentration 35 mmol/L.

b Missing values imputed as quintile 1.

c Rural location defined by community <10 000 residents.

d Standardized difference ≥0.1 compared to individualized dialysate bicarbonate.

e Number of visits/hospitalizations over the past year.

f Medication use was determined within the past 120 days for patients over 65 years of age or otherwise eligible for Ontario Drug Benefit coverage. Medication data available in 4861 (90%) patients.

g Values <6 cannot be reported due to ICES privacy regulations.

h Laboratory measurements were determined within the past year. The most recent outpatient value was used.

i Corrected for serum albumin using the formula: corrected calcium concentration = measured total calcium concentration in mmol/L + (0.02 * [40 g/L − serum albumin concentration in g/L]).

Abbreviations: AVF: arteriovenous fistula, AVG: arteriovenous graft, CVC: central venous catheter, BMI: body mass index, GN: glomerulonephritis, PKD: polycystic kidney disease, RVD: renovascular disease, PVD: peripheral vascular disease, COPD: chronic obstructive pulmonary disease, CABG: coronary artery bypass graft surgery, GI bleed: gastrointestinal bleed, PTH: parathyroid hormone.

### Dialysate Bicarbonate Categories and Outcomes

There were 62 816 serum bicarbonate measurements across 4854 patients. There were no significant differences in the mean concentration of pre-dialysis serum bicarbonate between the standardized (24.8 mmol/L) and individualized dialysate bicarbonate categories (25.0 mmol/L). The dialysate bicarbonate categories of 36 to 37 and ≥38 mmol/L had mean serum bicarbonate concentrations that were higher than the 35 mmol/L category (25.7, 26.0, and 24.1 mmol/L, respectively). All categories had a mean pre-dialysis serum bicarbonate within the normal range (Table 2). In the individualized category, 91% and 65% achieved a pre-dialysis serum bicarbonate ≥22 and ≥24 mmol/L, respectively, compared to 87% and 63% in the standardized category. Among the standardized dialysate bicarbonate categories, the proportion with a mean pre-dialysis serum bicarbonate ≥22 and ≥24 mmol/L, respectively, was 84% and 55% in the 35 mmol/L category, which was significantly lower than the 36 to 37 mmol/L category (97% and 82%) and the ≥38 mmol/L category (93% and 77%). On average, after adjustment, patients in the standardized category had a serum bicarbonate that was 0.25 (95% confidence interval [CI] = −0.93, 0.43) mmol/L lower than patients in the individualized category (*P* = .47). On average, after adjustment, patients in the 35, 36-37 and ≥38 mmol/L categories had a serum bicarbonate that was 0.60 (95% CI = −1.39, 0.19; *P* = .14) mmol/L lower, 0.06 (95% CI = −1.42, 1.55; *P* = .93) mmol/L higher, and 0.27 (95% CI = −0.72, 1.26; *P* = .59) mmol/L higher, respectively, compared to patients in the individualized category. On average, after adjustment, patients in the 36 to 37 and ≥38 mmol/L categories had a serum bicarbonate that was 0.70 (95% CI = −0.30, 1.70) mmol/L higher (*P* = .17) and 0.87 (95% CI = 0.14, 1.60) mmol/L higher (*P* = .02), respectively, compared to patients in the 35 mmol/L category (Tables 3 and 4).

**Table 2.** Mean (SD) Outcomes by Dialysate Bicarbonate Category.

	Individualized dialysate bicarbonate concentration	Standardized dialysate bicarbonate concentration			
		Categories of 35, 36-37, and ≥38 mmol/L all together	35 mmol/L	36-37 mmol/L	≥38 mmol/L
Pre-dialysis serum bicarbonate, (mmol/L)	25.0 (2.6)	24.8 (2.5)	24.1 (2.3)	25.7 (2.0)	26.0 (2.7)
Serum potassium, (mmol/L)	4.7 (0.5)	4.7 (0.5)	4.8 (0.5)	4.7 (0.4)	4.6 (0.5)
Serum calcium, (mmol/L)^a^	2.3 (0.2)	2.3 (0.2)	2.3 (0.2)	2.4 (0.2)	2.3 (0.2)
Serum albumin, (g/L)	37.5 (4.9)	37.0 (5.0)	36.9 (4.9)	32.8 (4.3)	39.0 (4.6)

a Corrected for serum albumin using the formula: corrected calcium concentration = measured total calcium concentration in mmol/L + (0.02 * [40 g/L − serum albumin concentration in g/L]).

Abbreviations: SD: standard deviation.

**Table 3.** Regression Coefficients for the Association of Dialysate Bicarbonate (Individualized Category as the Referent) With Outpatient Serum Bicarbonate (mmol/L).

Parameter	Unadjusted		Adjusted^a^	
	Estimate^b^ (95% CI)	*P*-value	Estimate^c^ (95% CI)	*P*-value
Individualized vs standardized^d^	−0.32 (−1.45, 0.80)	.58	−0.25 (−0.93, 0.43)	.47
Individualized vs 35 mmol/L	−0.83 (−2.01, 0.36)	.17	−0.60 (−1.39, 0.19)	.14
Individualized vs 36-37 mmol/L	0.22 (−2.02, 2.46)	.85	0.06 (−1.42, 1.55)	.93
Individualized vs ≥38 mmol/L	0.66 (−1.11, 2.44)	.47	0.27 (−0.72, 1.26)	.59

a Adjusted for age (continuous), sex, income quintile, rural status, vascular access, cause of end-stage kidney disease (ESKD), heart failure, peripheral vascular disease, COPD, coronary artery bypass surgery (CABG), gastrointestinal bleed, Charlson Comorbidity Index, number of hospitalizations, number of emergency room visits, number of primary care physician visits, number of cardiologist visits, smoking status, body mass index (BMI), and baseline hemoglobin, potassium, corrected calcium, albumin, parathyroid hormone, and serum bicarbonate.

b Intraclass correlation coefficient for individual patients 0.50 and for dialysis programs 0.16.

c Intraclass correlation coefficient for individual patients 0.36 and for dialysis programs 0.07.

d Categories of 35, 36 to 37, and ≥38 mmol/L all together.

Abbreviations: CI: confidence interval.

**Table 4.** Regression Coefficients for the Association of Dialysate Bicarbonate (35 mmol/L as the Referent) in the Standardized Group With Outpatient Serum Bicarbonate Concentration (mmol/L).

Parameter	Unadjusted		Adjusted^a^	
	Estimate^b^ (95% CI)	*P*-value	Estimate^c^ (95% CI)	*P*-value
35 mmol/L vs 36-37 mmol/L	1.06 (−0.75, 2.86)	.25	0.70 (−0.30, 1.70)	.17
35 mmol/L vs ≥38 mmol/L	1.48 (0.13, 2.83)	.0312	0.87 (0.14, 1.60)	.02

a Adjusted for age (continuous), sex, income quintile, rural status, vascular access, cause of end-stage kidney disease (ESKD), heart failure, peripheral vascular disease, COPD, coronary artery bypass surgery (CABG), gastrointestinal bleed, Charlson Comorbidity Index, number of hospitalizations, number of emergency room visits, number of primary care physician visits, number of cardiologist visits, smoking status, body mass index (BMI), and baseline hemoglobin, potassium, corrected calcium, albumin, parathyroid hormone, and serum bicarbonate.

b Intraclass correlation coefficient for individual patients 0.48 and for dialysis programs 0.12.

c Intraclass correlation coefficient for individual patients 0.34 and for dialysis programs 0.04.

Abbreviations: CI: confidence interval.

There were no significant differences between dialysate bicarbonate categories for serum potassium, corrected calcium, or albumin (Table 2 and Supplemental Tables 1-6).

Age, sex, or a history of COPD did not significantly modify the association between dialysate bicarbonate category and pre-dialysis serum bicarbonate concentration (Supplemental Table 7). An analysis restricted to individuals for which medication data were available (n = 4861), with additional adjustment performed for differences in medications, showed similar results to the primary analysis with respect to the association between dialysate bicarbonate category and the pre-dialysis serum bicarbonate concentration (Supplemental Tables 8 and 9).

## Discussion

In this retrospective cohort study that included 5414 patients receiving maintenance hemodialysis, we found no significant difference in the pre-dialysis serum bicarbonate concentration irrespective of whether an individualized or standardized approach to dialysis bicarbonate prescription was used. A higher dialysate bicarbonate concentration of ≥38 mmol/L, compared to 35 mmol/L, was associated with a statistically significant but modest increase in the mean pre-dialysis serum bicarbonate (0.9 mmol/L). Older age, sex, and a history of COPD did not modify the association between dialysate bicarbonate category and serum bicarbonate concentration. There were also no significant differences in the secondary outcomes of pre-dialysis potassium, corrected calcium, or serum albumin between dialysate bicarbonate categories.

Guidelines recommend normalizing the pre-dialysis serum bicarbonate based on evidence from small trials that suggests correcting metabolic acidosis (increase in bicarbonate ~5 mmol/L) has positive effects on nutrition, muscle mass, and bone metabolism.^5-8^ The intermittent nature of hemodialysis means that correction of metabolic acidosis needs to occur during the short time-frame of the dialysis session, and this correction must last until the next dialysis session. A primary focus on normalizing the pre-dialysis serum bicarbonate has resulted in the common use of high dialysate bicarbonate concentrations (ie, 35-40 mmol/L), especially in North America.^10^

We showed that individualization, compared to a standardized concentration, resulted in a modestly higher proportion of patients (4%) achieving a pre-dialysis serum bicarbonate ≥22 mmol/L but overall, did not result in a significant difference in mean pre-dialysis serum bicarbonate concentrations. Our data obtained from dialysis program directors on dialysate bicarbonate prescribing suggest that many centers that practiced individualization selected between only 2 dialysate bicarbonate concentrations, eg, 35 or 40 mmol/L, rather than using a broad range of dialysate bicarbonate concentrations. This practice may have limited the impact of individualization on pre-dialysis serum bicarbonate. Another potential explanation for the similar mean serum bicarbonate values between the individualized and standardized groups is that the impact of dialysate bicarbonate on pre-dialysis serum bicarbonate concentration is overestimated. Small studies with measures of both pre-dialysis and post-dialysis serum bicarbonate and pH show that large variations of the dialysate bicarbonate concentration between 30 and 40 mmol/L have little effect on the pre-dialysis pH (average increase of 0.02) and pre-dialysis bicarbonate concentration (average increase of 2 mmol/L).^11-15^ Similar to our findings, a prior large, international observational study showed that using an individualized approach increased the pre-dialysis bicarbonate by 0.2 mmol/L, whereas a standardized approach increased the pre-dialysis serum bicarbonate by 0.4 mmol/L for each 4 mmol/L increase in the dialysate bicarbonate concentration (interaction *P* = .7).^10^

Further supporting the limited impact of dialysate bicarbonate on pre-dialysis serum bicarbonate concentration, we found that a dialysate concentration of ≥38 mmol/L, compared to 35 mmol/L, was associated with a significantly higher mean serum bicarbonate concentration of only 0.9 (95% CI = 0.1-1.6) mmol/L upon adjustment; the clinical significance of this very modest increase is uncertain, even at the 1.6 mmol/L upper limit of the 95% CI. Similar to our results, the correlation between concentrations of dialysate bicarbonate and pre-dialysis serum bicarbonate was found to be weak (Spearman’s correlation coefficient = 0.09) in a prior large observational study of hemodialysis patients.^10^ Patient-related factors during the inter-dialytic period, such as protein and fluid intake, that also impact the pre-dialysis serum bicarbonate, likely mitigate the impact of the dialysate bicarbonate concentration on this outcome. For example, multiple studies show that the pre-dialysis serum bicarbonate concentration correlates inversely with measures of daily protein intake, serum albumin, and serum phosphate (a marker of higher dietary phosphorus).^3,4,21-27^

We found a higher proportion of patients achieved a pre-dialysis serum bicarbonate ≥22 mmol/L in the ≥38 mmol/L compared to the 35 mmol/L dialysate bicarbonate category (93% compared to 84%). However, this may be at the expense of inducing more post-dialysis alkalosis, a consequence of high dialysate bicarbonate concentration use that has been observed in prior studies.^8,11,28^ It is also important to note that all dialysate bicarbonate categories had a normal mean pre-dialysis serum bicarbonate (range = 24.1-26.0 mmol/L). This suggests that the mean post-dialysis serum bicarbonate concentration for all categories likely would be into the alkalotic range. A prior small study showed a mean post-dialysis pH of 7.51 and serum bicarbonate of 31.2 mmol/L with the use of a dialysate bicarbonate concentration of 35 mmol/L.^11^ The degree and duration of metabolic alkalosis is likely under-recognized as post-dialysis bloodwork is not routinely measured in the clinical setting.

Potential concerns with the use of very high dialysate bicarbonate concentrations were raised by a large cohort study using the Dialysis Outcomes and Practice Patterns Study (DOPPS) data, which showed that every 4 mmol/L rise in the dialysate bicarbonate concentration was associated with a higher risk of all-cause mortality (adjusted hazard ratio [HR] = 1.08, 95% CI = 1.01-1.15).^10^ This result could be due to confounding, but small studies demonstrate potential physiological, causal mechanisms supporting the observation. Post-dialysis metabolic alkalosis can lead to a lower concentration of serum potassium due to intracellular shifting,^29-31^ a longer corrected Q-T interval (which can increase the risk of cardiac arrhythmia),^32^ a higher drop in cardiac index^33^ during and post-dialysis, and more hypotension during dialysis.^30^ Other metabolic parameters affected by higher dialysate bicarbonate include lower ionized calcium^32^ and higher parathyroid hormone levels.^34,35^ Our study did not find any significant differences in pre-dialysis potassium or calcium. However, like serum bicarbonate, the dialysate bicarbonate concentration likely has the greatest impact on post-dialysis measures. We were unfortunately unable to study post-dialysis concentrations due to a lack of post-dialysis bloodwork routinely collected in Ontario, Canada. We also did not find any differences in serum albumin, a surrogate measure of nutrition, suggesting that the range of dialysate bicarbonate concentrations used in our study has a similar protective effect against the harms of metabolic acidosis on nutrition.

An important strength of our study is the large, generalizable cohort of patients receiving maintenance hemodialysis, along with access to laboratory data from almost the entire province of Ontario. However, our study has important limitations. First, we could not directly confirm that all laboratory measurements were pre-dialysis, but it is common practice in the dialysis center to only perform bloodwork prior to dialysis. We also limited outcomes to outpatient values to ensure that nearly all bloodwork would have been collected by a dialysis nurse prior to dialysis, and we excluded outpatient serum bicarbonate values that were biologically implausible (<10 or >45 mmol/L) from the analysis. The second limitation is that most patients received a dialysate bicarbonate concentration of 35 mmol/L. There were no dialysis programs in the standardized category in Ontario using concentrations <35 mmol/L, which limited the range of dialysate bicarbonate concentrations that we could examine. The third limitation is that we did not have data on prescribed dialysate bicarbonate concentrations for individual dialysis sessions, which may have led to some misclassification, and adherence to a practice of individualization could not be measured. Finally, there were some differences in baseline characteristics between the dialysate bicarbonate concentration categories. We performed adjustment, but residual confounding is possible. We also lacked data on dialysis-related variables, such as dialysis adequacy, for adjustment. It is, however, important to note the variation in dialysate bicarbonate practices across Ontario dialysis programs is primarily driven by historical practices of local nephrologists rather than by patient parameters.

In conclusion, we found that the pre-dialysis serum bicarbonate concentration was similar whether an individualized or standardized approach to prescribing dialysate bicarbonate was used. A dialysate bicarbonate concentration of ≥38 mmol/L, compared to 35 mmol/L, was associated with a statistically significant increase in the mean pre-dialysis serum bicarbonate concentration of less than 1 mmol/L, a difference of questionable clinical significance. The common practices of individualization of the dialysate bicarbonate concentration and the use of higher dialysate bicarbonate concentrations are both driven by the goal of normalizing the pre-dialysis serum bicarbonate concentration. However, these practices both appear to have limited impact on this measure and may be causing harm by inducing significant post-dialysis alkalosis and other post-dialysis electrolytes changes. Large multicenter randomized trials assessing clinically important outcomes are needed to determine the optimal dialysate bicarbonate practices and concentration for patient health.

## Supplemental Material

sj-docx-1-cjk-10.1177_20543581241256774 – Supplemental material for Association Between the Dialysate Bicarbonate and the Pre-dialysis Serum Bicarbonate Concentration in Maintenance Hemodialysis: A Retrospective Cohort Study Supplemental material, sj-docx-1-cjk-10.1177_20543581241256774 for Association Between the Dialysate Bicarbonate and the Pre-dialysis Serum Bicarbonate Concentration in Maintenance Hemodialysis: A Retrospective Cohort Study by Amber O. Molnar, Lauren Killin, Sarah Bota, Eric McArthur, Stephanie N. Dixon, Amit X. Garg, Claire Harris, Stephanie Thompson, Karthik Tennankore, Peter G. Blake, Clara Bohm, Jennifer MacRae and Samuel A. Silver in Canadian Journal of Kidney Health and Disease
