# Supplementary material for: Association Between the Dialysate Bicarbonate and the Pre-dialysis Serum Bicarbonate Concentration in Maintenance Hemodialysis: A Retrospective Cohort Study
Source: Can J Kidney Health Dis. 2024 May 30;11:20543581241256774. doi: 10.1177/20543581241256774 (PMC11141227; doi:10.1177/20543581241256774)
Supplement: sj-docx-1-cjk-10.1177_20543581241256774 – Supplemental material for Association Between the Dialysate Bicarbonate and the Pre-dialysis Serum Bicarbonate Concentration in Maintenance Hemodialysis: A Retrospective Cohort Study [file sj-docx-1-cjk-10.1177_20543581241256774.docx]

**Table of Contents**

Appendix A: REporting of studies Conducted using Observational Routinely collected health Data (RECORD) guidelines for observational studies

Appendix B: Databases and codes used

Supplemental Table 1: Regression coefficients for the association between the concentration of the dialysate bicarbonate and the outpatient concentration of serum potassium (mmol/L), with the individualized category serving as the referent group

Supplemental Table 2: Regression coefficients for the association of dialysate bicarbonate (35 mmol/L as the referent) in the standardized group with outpatient serum potassium concentration (mmol/L)

Supplemental Table 3: Regression coefficients for the association between the concentration of the dialysate bicarbonate and the outpatient concentration of serum calcium^a^ (mmol/L), with the individualized category serving as the referent group

Supplemental Table 4: Regression coefficients for the association of dialysate bicarbonate (35 mmol/L as the referent) in the standardized group with outpatient serum calcium^a^ concentration (mmol/L)

Supplemental Table 5: Regression coefficients for the association between the concentration of the dialysate bicarbonate and the outpatient concentration of serum albumin (g/L), with the individualized category serving as the referent group

Supplemental Table 6: Regression coefficients for the association of dialysate bicarbonate (35 mmol/L as the referent) in the standardized group with outpatient serum albumin concentration (g/L)

Supplemental Table 7: Adjusted regression coefficients for the association of serum bicarbonate with dialysate bicarbonate (mmol/L), subgroup analyses

Supplemental Table 8: Regression coefficients for the association between the concentration of the dialysate bicarbonate and the outpatient concentration of serum bicarbonate (mmol/L), with the individualized category serving as the referent group, analysis restricted to individuals with medication data (n=4,861)

Supplemental Table 9: Regression coefficients for the association of dialysate bicarbonate (35 mmol/L as the referent) in the standardized group with outpatient serum bicarbonate concentration (mmol/L), analysis restricted to individuals with medication data (n=4,861)

Supplemental Figure 1: Cohort Selection Flow Chart

**Appendix A: The RECORD statement**

|  | **Item No.** | **STROBE items** | **RECORD items** | **Location in manuscript where items are reported** |
| --- | --- | --- | --- | --- |
|  | 1 | (a) Indicate the study’s design with a commonly used term in the title or the abstract (b) Provide in the abstract an informative and balanced summary of what was done and what was found | RECORD 1.1: The type of data used should be specified in the title or abstract. When possible, the name of the databases used should be included.  RECORD 1.2: If applicable, the geographic region and timeframe within which the study took place should be reported in the title or abstract.  RECORD 1.3: If linkage between databases was conducted for the study, this should be clearly stated in the title or abstract. | Abstract; Methods |
| Background rationale | 2 | Explain the scientific background and rationale for the investigation being reported |  | Introduction |
| Objectives | 3 | State specific objectives, including any prespecified hypotheses |  | Introduction |
| Study Design | 4 | Present key elements of study design early in the paper |  | Methods; Design and Setting |
| Setting | 5 | Describe the setting, locations, and relevant dates, including periods of recruitment, exposure, follow-up, and data collection |  | Methods; Study Cohort |
| Participants | 6 | *(a) Cohort study* - Give the eligibility criteria, and the sources and methods of selection of participants. Describe methods of follow-up  *Case-control study* - Give the eligibility criteria, and the sources and methods of case ascertainment and control selection. Give the rationale for the choice of cases and controls  *Cross-sectional study* - Give the eligibility criteria, and the sources and methods of selection of participants  *(b) Cohort study* - For matched studies, give matching criteria and number of exposed and unexposed  *Case-control study* - For matched studies, give matching criteria and the number of controls per case | RECORD 6.1: The methods of study population selection (such as codes or algorithms used to identify subjects) should be listed in detail. If this is not possible, an explanation should be provided.  RECORD 6.2: Any validation studies of the codes or algorithms used to select the population should be referenced. If validation was conducted for this study and not published elsewhere, detailed methods and results should be provided.  RECORD 6.3: If the study involved linkage of databases, consider use of a flow diagram or other graphical display to demonstrate the data linkage process, including the number of individuals with linked data at each stage. | Methods; Study Cohort  Appendix B  N/A  Supplemental Fig.1 |
| Variables | 7 | Clearly define all outcomes, exposures, predictors, potential confounders, and effect modifiers. Give diagnostic criteria, if applicable. | RECORD 7.1: A complete list of codes and algorithms used to classify exposures, outcomes, confounders, and effect modifiers should be provided. If these cannot be reported, an explanation should be provided. | Methods; Dialysate Bicarbonate Groups and Outcomes  Appendix B |
| Data sources/ measurement | 8 | For each variable of interest, give sources of data and details of methods of assessment (measurement).  Describe comparability of assessment methods if there is more than one group |  | Methods; Data Sources  Appendix B |
| Bias | 9 | Describe any efforts to address potential sources of bias |  | Methods; Design and Setting |
| Study size | 10 | Explain how the study size was arrived at |  | Supplemental Figure 1 |
| Quantitative variables | 11 | Explain how quantitative variables were handled in the analyses. If applicable, describe which groupings were chosen, and why |  | Methods; Dialysate bicarbonate groups and outcomes  Methods; Statistical Analysis |
| Statistical methods | 12 | (a) Describe all statistical methods, including those used to control for confounding  (b) Describe any methods used to examine subgroups and interactions  (c) Explain how missing data were addressed  (d) *Cohort study* - If applicable, explain how loss to follow-up was addressed  *Case-control study* - If applicable, explain how matching of cases and controls was addressed  *Cross-sectional study* - If applicable, describe analytical methods taking account of sampling strategy  (e) Describe any sensitivity analyses |  | Methods; Statistical Analysis |
| Data access and cleaning methods |  | .. | RECORD 12.1: Authors should describe the extent to which the investigators had access to the database population used to create the study population.  RECORD 12.2: Authors should provide information on the data cleaning methods used in the study. | N/A |
| Linkage |  | .. | RECORD 12.3: State whether the study included person-level, institutional-level, or other data linkage across two or more databases. The methods of linkage and methods of linkage quality evaluation should be provided. | Methods; Design and Setting |
| Participants | 13 | (a) Report the numbers of individuals at each stage of the study (*e.g.*, numbers potentially eligible, examined for eligibility, confirmed eligible, included in the study, completing follow-up, and analysed)  (b) Give reasons for non-participation at each stage.  (c) Consider use of a flow diagram | RECORD 13.1: Describe in detail the selection of the persons included in the study (*i.e.,* study population selection) including filtering based on data quality, data availability and linkage. The selection of included persons can be described in the text and/or by means of the study flow diagram. | Supplemental Figure 1 |
| Descriptive data | 14 | (a) Give characteristics of study participants (*e.g.*, demographic, clinical, social) and information on exposures and potential confounders  (b) Indicate the number of participants with missing data for each variable of interest  (c) *Cohort study* - summarise follow-up time (*e.g.*, average and total amount) |  | Table 1:Baseline characteristics  Statistical analysis |
| Outcome data | 15 | *Cohort study* - Report numbers of outcome events or summary measures over time  *Case-control study* - Report numbers in each exposure category, or summary measures of exposure  *Cross-sectional study* - Report numbers of outcome events or summary measures |  | Results; Dialysate Bicarbonate Groups and Outcomes |
| Main results | 16 | (a) Give unadjusted estimates and, if applicable, confounder-adjusted estimates and their precision (e.g., 95% confidence interval). Make clear which confounders were adjusted for and why they were included  (b) Report category boundaries when continuous variables were categorized  (c) If relevant, consider translating estimates of relative risk into absolute risk for a meaningful time period |  | Results; Dialysate Bicarbonate Groups and Outcomes  Table 2: Outcomes by dialysate bicarbonate group  Table 3: Regression Coefficients for the association of serum bicarbonate with dialysate bicarbonate, individualized group as the referent  Table 4: Regression Coefficients for the association of serum bicarbonate with dialysate bicarbonate, Dialysate concentration 35 mmol/L group as the referent  Supplemental tables 1-6 |
| Other analyses | 17 | Report other analyses done—e.g., analyses of subgroups and interactions, and sensitivity analyses |  | Supplemental table 7 |
| Key results | 18 | Summarise key results with reference to study objectives |  | Discussion |
| Limitations | 19 | Discuss limitations of the study, taking into account sources of potential bias or imprecision. Discuss both direction and magnitude of any potential bias | RECORD 19.1: Discuss the implications of using data that were not created or collected to answer the specific research question(s). Include discussion of misclassification bias, unmeasured confounding, missing data, and changing eligibility over time, as they pertain to the study being reported. | Discussion |
| Interpretation | 20 | Give a cautious overall interpretation of results considering objectives, limitations, multiplicity of analyses, results from similar studies, and other relevant evidence |  | Discussion |
| Generalisability | 21 | Discuss the generalisability (external validity) of the study results |  | Discussion |
| Funding | 22 | Give the source of funding and the role of the funders for the present study and, if applicable, for the original study on which the present article is based |  | Acknowledgments |
| Accessibility of protocol, raw data, and programming code |  | .. | RECORD 22.1: Authors should provide information on how to access any supplemental information such as the study protocol, raw data, or programming code. | Data Sharing Statement |

**Appendix B: Databases and Codes Used**

| **Variable** | Source | Specific definition or codes |
| --- | --- | --- |
| **Baselines** |  |  |
| **Age** | RPDB | BDATE |
| **Sex** | RPDB | SEX |
| **Income quintile** | RPDB |  |
| **Rurality** | RPDB |  |
| **Time on dialysis** | CORR | TREATMENT_CODE, TREATMENT_DATE. Look back to start of CORR for any dialysis |
| **Arteriovenous fistula access** | ORRS | PRIMARYACCESSTYPECD/INITIALACCESSUSED= “5” |
| **Central venous catheter access** | ORRS  OHIP | PRIMARYACCESSTYPECD/INITIALACCESSUSED= (“1”, “2”, “3”, “4”)  FEECODE R848, G099, G312, G324, G327, G336 |
| **Arteriovenous graft access** | ORRS | PRIMARYACCESSTYPECD / INITIALDIALYSISACCESSUSED = (“6”) |
| **Smoking at dialysis initiation** | CORR  ORRS | CURRENT_SMOKER_FLAG=”Y”  SMOKECD=”Y” |
| **Body Mass Index at dialysis initiation** | ORRS | BMI=(INITIAL_WEIGHT/((INITIAL_HEIGHT)/100)^2^)) |
| **Primary cause of End Stage Kidney Disease** | ORRS  CORR | PRDTYPECD  Primary_Diagnosis_Kidney. To supplement missing or UNKNOWN in ORRS. |
| **Hospitalizations in previous year** | DAD | ADMDATE |
| **Emergency Department visits in previous year** | NACRS | REGDATE |
| **Family physician visits in previous year** | OHIP  CPDB | SPEC= “00”  FEECODE A001, A003-006, A008, A901, A905, A912, C912, K024, K025, Q525, W872, W912 |
| **Cardiologist visits in previous year** | OHIP  CPDB | SPEC= “60” |
| **Charlson comorbidity score** | DAD  SDS | Exclude the renal component from score. |
| **History of atrial fibrillation/flutter** | DAD  NACRS  OHIP | ICD10 I48  DXCODE 427 |
| **History of heart failure** | OHIP  DAD  SDS  NACRS | FEECODE R701, R702, Z429  DXCODE 428  ICD10 I099, I420, I425-29, I43, I500, I501, I509, I255, J81  CCI 1HP53, 1HP55, 1HZ53GRFR, 1HZ53LAFR, 1HZ53SYFR |
| **History of peripheral vascular disease** | OHIP  DAD  SDS  NACRS | FEECODE R780, R787, R797, R804, R809, R825, R936, R783, R784, R785, E626, R814, R786, R937, R860, R861, R855, R856, R933, R934, R791, E672, R794, R813, R867, E649  ICD10 I700, I702, I708, I709, I731, I738, I739, K551  CCI 1KA76, 1KA50, 1KE76, 1KG57, 1KG76MIXXA, 1KG76MIXXN, 1KG76MIXXQ, 1KG87, 1IA87LA, 1IB87LA, 1IC87LA, 1ID87, 1KA87LA, 1KE57 |
| **History of ischemic stroke** | DAD  SDS | Subgroup of stroke/TIA definition above.  ICD10 H341, I630, I631, I632, I633, I634, I635, I638, I639, I64 |
| **History of diabetes** | ODD | ICES ODD database |
| **History of Chronic Obstructive Pulmonary Disease** | COPD | ICES COPD database |
| **History of Coronary artery bypass graft surgery** | DAD  SDS  OHIP | FEECODE R742, R743, E654, E645, E652, E646  CCI 1IJ76 |
| **History of GI bleed** | DAD  NACRS | ICD10 K250, K252, K254, K256, K260, K262, K264, K266, K270, K272, K274, K276, K280, K282, K284, K286, K920, K921, K922, K5220, K226, I850 |
| **History of major cancer (excluding skin cancer)** | OHIP  DAD  SDS  NACRS | DXCODE 150, 154, 155, 157, 162, 174, 175, 183, 185, 203, 204, 205, 206, 207, 208  ICD10 971, 980, 982, 984, 98503, 986, 98703, 98803, 989, 99003, 99103, 993, C15, C18, C19, C20, C22, C25, C34, C50, C56, C61, C82, C83, C85, C91, C92, C93, C94, C95, D00, D010, D011, D012, D022, D05, D075 |
| **Medications** | ODB |  |
| **Hemoglobin lab test** | OLIS  ORRS | LOINC 718-7, 20509-6 |
| **Serum albumin lab test and value** | OLIS  ORRS | LOINC 1751-7 |
| **Parathyroid hormone lab test and value** | OLIS | LOINC 14866-8, 47178-9, 47093-0, 47180-5 |
| **Serum potassium lab test and value** | OLIS | LOINC 22760-3, 2823-3, 39789-3, 6298-4 |
| **Total calcium lab test and value** | OLIS | LOINC corrected calcium 18281-6, 29265-5  LOINC uncorrected calcium 1996-8, 2000-8, 42857-3  Used following formula to convert uncorrected calcium to corrected calcium values: Corrected [Ca] mmol/l = Measured total [Ca] mmol/l + (0.02 x (40.0 g/l - [alb] g/l) |
| **Serum bicarbonate lab test and value** | OLIS | LOINC 1963-8, 14627-4, 1962-0, 1959-6, 19232-8, 1961-2, 2027-1, 2028-9 |
| **Outcomes** | | |
| **Serum bicarbonate concentration^†*^** | OLIS | LOINC 1963-8, 14627-4, 1962-0, 1959-6, 19232-8, 1961-2, 2027-1, 2028-9  Pull all outpatient serum bicarbonate values in the follow up period. |
| **Serum potassium concentration^†*^** | OLIS | LOINC 22760-3, 2823-3, 39789-3, 6298-4 |
| **Total calcium concentration^†*^** | OLIS | LOINC corrected calcium 18281-6, 29265-5  LOINC uncorrected calcium 1996-8, 2000-8, 42857-3  Used following formula to convert uncorrected calcium to corrected calcium values: Corrected [Ca] mmol/l = Measured total [Ca] mmol/l + (0.02 x (40.0 g/l - [alb] g/l) |
| **Serum albumin concentration^†*^** | OLIS | LOINC 1751-7 |
| **Censoring Events** |  |  |
| **Death** | ORRS  RPDB | TREATMENTCHANGECD= “D” |
| **Transfer out of index dialysis program** | ORRS | TRANSFERHOSPITALCD= “TO” |
| **Permanent stop to ICHD** | ORRS  OHIP | TREATMENTCHANGECD in “TX”, “W”, “R’  FEECODE S435, S434 |
| **Emigration** | ORRS  RPDB | TREATMENTCHANGECD= “X”  DOLC |

**†**Lab values were restricted to outpatient lab values; only lab values captured outside of admission and discharge dates in DAD and NACRS were included. Lab values were restricted to values within a stable, outpatient reference range for the respective lab test.

*Patients were followed for outcomes until one of the following censoring events were reached: maximum follow up date (March 31, 2021), death, transfer out of index dialysis program, permanent stop to ICHD (recover, withdrawal, transfer to non-ICHD, ICHD short daily or ICHD nocturnal modalities, transplant), emigration.

Abbreviations: RPDB: Registered Persons Database, CORR: Canadian Organ Replacement Register, ORRS: Ontario Renal Reporting System, OHIP: Ontario Health Insurance Program, DAD: Discharge Abstract Database, NACRS: National Ambulatory Care Reporting System, CPDB: Canadian Physician Database, SDS: Same Day Surgery, ODD: ICES-Derived Ontario Diabetes Database, COPD: ICES-Derived Chronic Obstructive Pulmonary Disease Database, OLIS: Ontario Lab Information System. ODB: Ontario Drug Benefit Database. ODB contains records of all outpatient prescriptions dispensed to patients over 65 years of age with public drug coverage with an error rate <1%.

**Supplemental Table 1: Regression coefficients for the association between the concentration of the dialysate bicarbonate and the outpatient concentration of serum potassium (mmol/L), with the individualized category serving as the referent group**

| **Parameter** | **Unadjusted Estimate**  **(95% CI)** | **P-value** | **Adjusted^a^**  **Estimate**  **(95% CI)** | **P-value** |
| --- | --- | --- | --- | --- |
| Individualized vs. standardized | 0.01  (-0.15, 0.16) | 0.94 | 0.01  (-0.08, 0.09) | 0.90 |
| Individualized vs. 35 mmol/L | 0.03  (-0.13, 0.20) | 0.68 | 0.03  (-0.06, 0.12) | 0.55 |
| Individualized vs. 36-37 mmol/L | 0.06  (-0.24, 0.35) | 0.71 | 0.01  (-0.12, 0.13) | 0.91 |
| Individualized vs. ≥38 mmol/L | -0.08  (-0.34, 0.17) | 0.51 | -0.05  (-0.16, 0.06) | 0.40 |

^a^Adjusted for age (continuous), sex, income quintile, rural status , vascular access, cause of end stage kidney disease (ESKD), heart failure, peripheral vascular disease, COPD, coronary artery bypass surgery (CABG), gastrointestinal bleed, Charlson Comorbidity Index, number of hospitalizations , number of emergency room visits, number of primary care physician visits, number of cardiologist visits, smoking status, body mass index (BMI), and baseline hemoglobin, potassium, corrected calcium, albumin, parathyroid hormone and serum bicarbonate.

**Supplemental Table 2: Regression coefficients for the association of dialysate bicarbonate (35 mmol/L as the referent) in the standardized group with outpatient serum potassium concentration (mmol/L)**

| **Parameter** | **Unadjusted Estimate**  **(95% CI)** | **P-value** | **Adjusted^a^ Estimate**  **(95% CI)** | **P-value** |
| --- | --- | --- | --- | --- |
| 35 mmol/L vs. 36-37 mmol/L | 0.02  (-0.28, 0.33) | 0.89 | 0.02  (-0.18, 0.21) | 0.86 |
| 35 mmol/L vs. ≥38 mmol/L | -0.12  (-0.35, 0.11) | 0.30 | -0.07  (-0.21, 0.07) | 0.32 |

^a^Adjusted for age (continuous), sex, income quintile, rural status , vascular access, cause of end stage kidney disease (ESKD), heart failure, peripheral vascular disease, COPD, coronary artery bypass surgery (CABG), gastrointestinal bleed, Charlson Comorbidity Index, number of hospitalizations , number of emergency room visits, number of primary care physician visits, number of cardiologist visits, smoking status, body mass index (BMI), and baseline hemoglobin, potassium, corrected calcium, albumin, parathyroid hormone and serum bicarbonate.

**Supplemental Table 3: Regression coefficients for the association between the concentration of the dialysate bicarbonate and the outpatient concentration of serum calcium^a^ (mmol/L), with the individualized category serving as the referent group**

| **Parameter** | **Unadjusted Estimate**  **(95% CI)** | **P-value** | **Adjusted^b^ Estimate**  **(95% CI)** | **P-value** |
| --- | --- | --- | --- | --- |
| Individualized vs. standardized | -0.03  (-0.07, 0.00) | 0.08 | -0.04  (-0.07, -0.00) | 0.03 |
| Individualized vs. 35 mmol/L | -0.04  (-0.08, 0.01) | 0.11 | -0.04  (-0.07, -0.00) | 0.05 |
| Individualized vs. 36-37 mmol/L | -0.05  (-0.13, 0.03) | 0.22 | -0.06  (-0.11, -0.00) | 0.04 |
| Individualized vs. ≥38 mmol/L | -0.02  (-0.08, 0.04) | 0.50 | -0.03  (-0.08, 0.02) | 0.24 |

^a^Corrected for serum albumin using the formula: corrected calcium concentration = measured total calcium concentration in mmol/L + [0.02* (40 g/L-serum albumin concentration in g/L)].

^b^Adjusted for age (continuous), sex, income quintile, rural status , vascular access, cause of end stage kidney disease (ESKD), heart failure, peripheral vascular disease, COPD, coronary artery bypass surgery (CABG), gastrointestinal bleed, Charlson Comorbidity Index, number of hospitalizations , number of emergency room visits, number of primary care physician visits, number of cardiologist visits, smoking status, body mass index (BMI), and baseline hemoglobin, potassium, corrected calcium, albumin, parathyroid hormone and serum bicarbonate.

**Supplemental Table 4: Regression coefficients for the association of dialysate bicarbonate (35 mmol/L as the referent) in the standardized group with outpatient serum calcium^a^ concentration (mmol/L)**

| **Parameter** | **Unadjusted Estimate**  **(95% CI)** | **P-value** | **Adjusted^b^ Estimate**  **(95% CI)** | **P-value** |
| --- | --- | --- | --- | --- |
| 35 mmol/L vs. 36-37 mmol/L | -0.02  (-0.08, 0.05) | 0.64 | -0.00  (-0.07, 0.06) | 0.89 |
| 35 mmol/L vs. ≥38 mmol/L | 0.01  (-0.03, 0.06) | 0.56 | 0.01  (-0.04, 0.05) | 0.78 |

^a^Corrected for serum albumin using the formula: corrected calcium concentration = measured total calcium concentration in mmol/L + [0.02* (40 g/L-serum albumin concentration in g/L)].

^b^Adjusted for age (continuous), sex, income quintile, rural status , vascular access, cause of end stage kidney disease (ESKD), heart failure, peripheral vascular disease, COPD, coronary artery bypass surgery (CABG), gastrointestinal bleed, Charlson Comorbidity Index, number of hospitalizations , number of emergency room visits, number of primary care physician visits, number of cardiologist visits, smoking status, body mass index (BMI), and baseline hemoglobin, potassium, corrected calcium, albumin, parathyroid hormone and serum bicarbonate.

**Supplemental Table 5: Regression coefficients for the association between the concentration of the dialysate bicarbonate and the outpatient concentration of serum albumin (g/L), with the individualized category serving as the referent group**

| **Parameter** | **Unadjusted Estimate**  **(95% CI)** | **P-value** | **Adjusted^a^ Estimate**  **(95% CI)** | **P-value** |
| --- | --- | --- | --- | --- |
| Individualized vs. standardized | -0.09  (-2.15, 1.96) | 0.93 | 0.19  (-0.84, 1.21) | 0.72 |
| Individualized vs. 35 mmol/L | -0.07  (-2.24, 2.10) | 0.95 | 0.31  (-0.85, 1.47) | 0.60 |
| Individualized vs. 36-37 mmol/L | -2.98  (-7.42, 1.47) | 0.19 | -1.59  (-3.32, 0.15) | 0.07 |
| Individualized vs. ≥38 mmol/L | 1.26  (-1.67, 4.19) | 0.40 | 0.50  (-0.73, 1.72) | 0.43 |

^a^Adjusted for age (continuous), sex, income quintile, rural status , vascular access, cause of end stage kidney disease (ESKD), heart failure, peripheral vascular disease, COPD, coronary artery bypass surgery (CABG), gastrointestinal bleed, Charlson Comorbidity Index, number of hospitalizations , number of emergency room visits, number of primary care physician visits, number of cardiologist visits, smoking status, body mass index (BMI), and baseline hemoglobin, potassium, corrected calcium, albumin, parathyroid hormone and serum bicarbonate.

**Supplemental Table 6: Regression coefficients for the association of dialysate bicarbonate (35 mmol/L as the referent) in the standardized group with outpatient serum albumin concentration (g/L)**

| **Parameter** | **Unadjusted Estimate**  **(95% CI)** | **P-value** | **Adjusted^a^**  **Estimate**  **(95% CI)** | **P-value** |
| --- | --- | --- | --- | --- |
| 35 mmol/L vs. 36-37 mmol/L | -2.96  (-6.29, 0.37) | 0.08 | -1.87  (-3.72, -0.03) | 0.05 |
| 35 mmol/L vs. ≥38 mmol/L | 1.36  (-1.14, 3.85) | 0.29 | 0.07  (-1.31, 1.45) | 0.92 |

^a^Adjusted for age (continuous), sex, income quintile, rural status , vascular access, cause of end stage kidney disease (ESKD), heart failure, peripheral vascular disease, COPD, coronary artery bypass surgery (CABG), gastrointestinal bleed, Charlson Comorbidity Index, number of hospitalizations , number of emergency room visits, number of primary care physician visits, number of cardiologist visits, smoking status, body mass index (BMI), and baseline hemoglobin, potassium, corrected calcium, albumin, parathyroid hormone and serum bicarbonate.

**Supplemental Table 7: Adjusted^a^ regression coefficients for the association of serum bicarbonate with dialysate bicarbonate (mmol/L), subgroup analyses**

| **Strata** | | **Individualized vs. standardized^b^** | **35 mmol/L vs. 36-37 mmol/L** | **35 mmol/L vs. ≥38 mmol/L** |
| --- | --- | --- | --- | --- |
|  |  | **Estimate (95% CI)** | **Estimate (95% CI)** | **Estimate (95% CI)** |
| Age | <65 | -0.29 (-1.05, 0.47) | 0.77 (-0.45, 1.98) | 0.72 (0.44, -0.15) |
|  | ≥65-80 | -0.27 (-0.96, 0.43) | 0.48 (-0.59, 1.56) | 0.91 (0.16, 1.67) |
|  | >80 | -0.18 (-0.80, 0.45) | 0.79 (-0.14, 1.71) | 0.96 (0.27, 1.65) |
| Sex | Female | -0.32 (-1.05, 0.41) | 0.86 (-0.24, 1.97) | 0.80 (0.05, 1.55) |
|  | Male | -0.20 (-0.89, 0.49) | 0.66 (-0.38, 1.70) | 0.95 (0.18, 1.71) |
| COPD | Yes | -0.47 (-1.18, 0.24) | 0.57 (-0.60, 1.74) | 0.94 (0.08, 1.80) |
|  | No | -0.14 (-0.83, 0.55) | 0.77 (-0.23, 1.76) | 0.83 (0.12, 1.53) |

^a^Adjusted for age (continuous), sex, income quintile, rural status , vascular access, cause of end stage kidney disease (ESKD), heart failure, peripheral vascular disease, COPD, coronary artery bypass surgery (CABG), gastrointestinal bleed, Charlson Comorbidity Index, number of hospitalizations , number of emergency room visits, number of primary care physician visits, number of cardiologist visits, smoking status, body mass index (BMI), and baseline hemoglobin, potassium, corrected calcium, albumin, parathyroid hormone and serum bicarbonate.

^b^Categories of 35, 36-37 and ≥38 mmol/L all together

Abbreviations: CI: confidence interval, COPD: chronic obstructive pulmonary disease

**Supplemental Table 8: Regression coefficients for the association between the concentration of the dialysate bicarbonate and the outpatient concentration of serum bicarbonate (mmol/L), with the individualized category serving as the referent group, analysis restricted to individuals with medication data (n=4,861)**

| **Parameter** | **Unadjusted** | | **Adjusted^a^** | |
| --- | --- | --- | --- | --- |
|  | **Estimate**  **(95% CI)** | **P-value** | **Estimate**  **(95% CI)** | **P-value** |
| Individualized vs. standardized^b^ | -0.30  (-1.42, 0.82) | 0.60 | -0.25  (-0.89, 0.40) | 0.45 |
| Individualized vs. 35 mmol/L | -0.81  (-1.98, 0.37) | 0.18 | -0.59  (-1.35, 0.17) | 0.13 |
| Individualized vs. 36-37 mmol/L | 0.24  (-2.00, 2.48) | 0.83 | 0.06  (-1.36, 1.47) | 0.94 |
| Individualized vs. ≥38 mmol/L | 0.69  (-1.08, 2.47) | 0.44 | 0.23  (-0.70, 1.16) | 0.63 |

^a^Adjusted for age (continuous), sex, income quintile, rural status , vascular access, cause of end stage kidney disease (ESKD), heart failure, peripheral vascular disease, COPD, coronary artery bypass surgery (CABG), gastrointestinal bleed, Charlson Comorbidity Index, number of hospitalizations , number of emergency room visits, number of primary care physician visits, number of cardiologist visits, smoking status, body mass index (BMI), and baseline hemoglobin, potassium, corrected calcium, albumin, parathyroid hormone, serum bicarbonate, and baseline medication use (diuretics, calcium carbonate, sevelamer, proton pump inhibitors, statins, beta-blockers, angiotensin converting enzyme inhibitors, angiotensin receptor blockers, oral anticoagulants, alpha blockers, calcium channel blockers, nitrates, hydralazine).

^b^Categories of 35, 36-37 and ≥38 mmol/L all together

Abbreviations: CI: confidence interval

**Supplemental Table 9: Regression coefficients for the association of dialysate bicarbonate (35 mmol/L as the referent) in the standardized group with outpatient serum bicarbonate concentration (mmol/L), analysis restricted to individuals with medication data (n=4,861)**

| **Parameter** | **Unadjusted** | | **Adjusted^a^** | |
| --- | --- | --- | --- | --- |
|  | **Estimate**  **(95% CI)** | **P-value** | **Estimate**  **(95% CI)** | **P-value** |
| 35 mmol/L vs. 36-37 mmol/L | 1.05  (-0.71, 2.82) | 0.24 | 0.70  (-0.25, 1.64) | 0.15 |
| 35 mmol/L vs. ≥38 mmol/L | 1.49  (0.18, 2.81) | 0.03 | 0.81  (0.12, 1.50) | 0.02 |

^a^Adjusted for age (continuous), sex, income quintile, rural status , vascular access, cause of end stage kidney disease (ESKD), heart failure, peripheral vascular disease, COPD, coronary artery bypass surgery (CABG), gastrointestinal bleed, Charlson Comorbidity Index, number of hospitalizations , number of emergency room visits, number of primary care physician visits, number of cardiologist visits, smoking status, body mass index (BMI), and baseline hemoglobin, potassium, corrected calcium, albumin, parathyroid hormone, serum bicarbonate, and baseline medication use (diuretics, calcium carbonate, sevelamer, proton pump inhibitors, statins, beta-blockers, angiotensin converting enzyme inhibitors, angiotensin receptor blockers, oral anticoagulants, alpha blockers, calcium channel blockers, nitrates, hydralazine).

Abbreviations: CI: confidence interval

| **Supplemental Figure 1**: **Cohort Selection Flow Chart**  5,652 Individuals receiving maintenance in-centre hemodialysis at the same regional dialysis program for at least 120 days by April 1, 2020  232 Individuals excluded due to receiving dialysis treatment at a regional dialysis program that does not capture laboratory data  5,414 Ontario adults receiving maintenance hemodialysis included in the analysis  5,646 Individuals with valid health identification number, age, sex, alive on index date, Ontario resident, age >18 years old  6 Individuals excluded due to invalid health identification number, age, or sex, death on or before index date, non-Ontario resident or age <18 years old |
| --- |
|  |
|  |
|  |
|  |
|  |
|  |
|  |
|  |
|  |
|  |
|  |
|  |
|  |
|  |
|  |
|  |
|  |
|  |
|  |
|  |
|  |
|  |
|  |
|  |
|  |
|  |
|  |
|  |
|  |
|  |
|  |
|  |
|  |
|  |
|  |
|  |

|  |
| --- |
|  |
